# Two Homologous Putative Protein Tyrosine Phosphatases, OsPFA-DSP2 and AtPFA-DSP4, Negatively Regulate the Pathogen Response in Transgenic Plants

**DOI:** 10.1371/journal.pone.0034995

**Published:** 2012-04-13

**Authors:** Hanjie He, Jianbin Su, Shengying Shu, Yang Zhang, Ying Ao, Bing Liu, Dongru Feng, Jinfa Wang, Hongbin Wang

**Affiliations:** Key Laboratory of Gene Engineering of Ministry of Education, State Key Laboratory of Biocontrol and Guangdong Key Laboratory of Plant Resources, School of Life Sciences, Sun Yat-sen University, Guangzhou, People's Republic of China; University of South Florida College of Medicine, United States of America

## Abstract

Protein phosphatases, together with protein kinases, regulate protein phosphorylation and dephosphorylation, and play critical roles in plant growth and biotic stress responses. However, little is known about the biological functions of plant protein tyrosine dual-specificity phosphatase (PFA-DSP) in biotic stresses. Here, we found that *OsPFA-DSP2* was mainly expressed in calli, seedlings, roots, and young panicles, and localized in cytoplasm and nucleus. Ectopic overexpression of *OsPFA-DSP2* in rice increased sensitivity to *Magnaporthe grisea* (*M. grisea* Z1 strain), inhibited the accumulation of hydrogen peroxide (H_2_O_2_) and suppressed the expression of pathogenesis-related (*PR*) genes after fungal infection. Interestingly, transgenic Arabidopsis plants overexpressing *AtPFA-DSP4*, which is homologous to *OsPFA-DSP2*, also exhibited sensitivity to *Pseudomonas syringae* pv. *tomato* DC3000 (*Pst* DC3000), reduced accumulation of H_2_O_2_ and decreased photosynthesic capacity after infection compared with Col-0. These results indicate that OsPFA-DSP2 and AtPFA-DSP4 act as negative regulators of the pathogen response in transgenic plants.

## Introduction

Plants face a variety of biotic stresses in nature, including bacteria and fungi, which can strongly affect growth and production [1]. In response to these biotic stresses, plants have evolved many defense mechanisms [2]. In one such defense mechanism, reactive oxygen species (ROS) (H_2_O_2_, especially) play a central role in plants by controlling many biological processes, including gene expression, activation of transcription factors, redox balance, programmed cell death (PCD) and regulation of the mitogen-activated protein kinase (MAPK) pathway [3], [4], [5], [6]. Several studies have reported that high levels of ROS lead to the oxidative destruction of cells, but moderate levels can act as signaling molecules to regulate plant growth and the biotic stress response [7]. H_2_O_2_ is an important component of ROS; endogenous and exogenous H_2_O_2_ directly or indirectly killed pathogen cells or inhibited their growth, penetration and proliferation at the infection site by inducing PCD [3], [8], [9].

Reversible protein phosphorylation, mediated through the MAPK signalling cascade, plays a key role in determining the response to many external stimuli in plants, including biotic stresses [10], [11]. The process of reversible phosphorylation is controlled by a balance between the activities of protein kinases and protein phosphatases *in vivo* [12]. Plant MAPKs are phosphorylated (and activated) by a series of substrate-specific kinases (e.g., MAPKK, MAPKK). MAPKs are dephosphorylated, and hence deactivated, by dual-specificity MAP kinase phosphatases (DSPs) [12], [13]. The study by Asai et al. [14] reported the identification of the components in a MAPK signalling pathway in Arabidopsis. These authors demonstrated that in response to the flagellin-derived peptide flg22, AtMEKK1 (a MAPKKKs) activates AtMKK4 and AtMKK5 (two MAPKKs), which in turn activate the functionally redundant MAPKs AtMPK3 and AtMPK6. This cascade results in transcription of defense-related genes, and was shown to play an important role in resistance to both bacterial and fungal pathogen [14]. More recent studies have shown that a transcription factor in *Nicotiana benthamiana*, WRKY8, is phosphylated by several MAPKs (SIPK, NT4 and WIPK), which in turn results in transcription of defense-related genes, and also that phosphorylation of pathogen-inducible WRKY33 by MPK3/MPK6 in Arabidopsis is important for activating genes involved in phytoalexin biosynthesis [15], [16]. Phosphatases as well as protein kinases play critical roles in plant biotic stress. For example, the rice protein phosphatase XB15 (belonging to the PP2C subfamily), phosphorylated and inactivated protein kinase XA21, negatively regulating XA21-mediated innate immunity [17]. AtMKP2, a MAPK phosphatase, interacts differentially with AtMPK3 and AtMPK6 in the negative regulation of specific defense responses [18], [19]. Similarly, knockout mutants in *AtMKP1* displayed elevated resistance to *Pst* DC3000 by regulating AtMPK6 activity, again suggesting that phosphatases play key roles in the biotic stresses response [20].

Based on substrate specificity, protein tyrosine phosphatases (PTPs) can be divided into many groups, such as those that utilize phosphoproteins, lipids, deoxyribonucleic acids and carbohydrates [21]. In land plants, the lipid phosphatases are classified into three groups: tumor suppressor phosphatase and tension homologue deleted in chromosome 10 (PTEN), myotubular myopathy related protein (MTMR) and plant and fungal atypical dual-specificity phosphatases (PFA-DSP) [22]. The PFA-DSP subfamily is found in land plants and fungi, and uses phosphatidylinositol as substrate [22]. We previously demonstrated that OsPFA-DSP1 is an active tyrosine-specific phosphatase, and that it acts as a negative regulator in the abiotic stress response [23]. In addition, the AtPFA-DSP1 protein from *Arabidopsis thaliana* has been shown to possess phosphatase activity [24]. However, the biological function of PFA-DSPs in the biotic stress response is unknown. Here, we used overexpression and RNA-interference knockdown of *OsPFA-DSP2* and *AtPFA-DSP4* in transgenic rice and Arabidopsis plants to analyse their biological function during biotic stress. We also assayed the expression patterns and subcellular localization of OsPFA-DSP2, and used genetic techniques to study its biological functions. Our results indicate that *OsPFA-DSP2* and *AtPFA-DSP4* negatively regulate the host response to a fungal (*M. grisea*) and a bacterial (*Pst* DC3000) pathogen.

## Materials and Methods

### Generation of transgenic plants

The overexpression construct for *OsPFA-DSP2* (Loc_Os02g53160) from *Oryza sativa* ssp. *japonica* cv. Nipponbare was created by inserting a complete cDNA sequence into the vector pCXUN-flag, which contained a maize (*Zea mays*) ubiquitin gene promoter [25]. *OsPFA-DSP2* primer sequences are as follows: (sense primer) 5′-ATGCAGCTGGAGATTTCG-3′ and (antisense primer) 5′-TTAACACTGTGAGGCCGTC-3′. The *OsPFA-DSP2* interference construct was created by inserting a genomic sequence fragment (containing 125 bp of the 3′UTR and 125 bp from the translation stop codon) into the binary vector pCAMBIA1301,which carries the cauliflower mosaic virus (CaMV) 35S promoter. The recombinant plasmids were introduced into *Agrobacterium tumefaciens* strain EHA105 by electroporation. *Agrobacterium tumefaciens* mediated transformation were performing using calli derived from mature embryos of *japonica* line Nipponbare, according to the published protocol [26]. The primer sequences for the interference vector were as follows: sense fragment, 5′-GGTAAGCTTCGAATGTTTTTTCATATCCGGTC-3′ and 5′-GCACCATGGGATGGATACTTTTATGAGGATAA-3′, with *Hind*III and *Nco*I sites (underlined); antisense fragment, 5′-CGCACTAGTTGTATTTTGATGGATACTTTTATGAG-3′ and 5′-ATTAGGCCTCCGAACCGAATGTTTTTTCAT-3′, with *Spe*I and *Stu*I sites (underlined), respectively. Transgenic rice seedlings were screened on 1/2 MS solid medium containing 50 µg/mL hygromycin.

Using gene-specific primers, the full-length cDNA sequence of *AtPFA-DSP4* (At4g03960) from Col-0 was amplified and cloned into vector pEN1A between the *Sal*I and *EcoR*V sites with primers 5′-AGTCGACATGACGTTAGAGAGTTAC-3′ and 5′-CGGATATCTCAGTAATCAATAGTATT-3′. The resulting vector was used to transfer the *AtPFA-DSP4* gene to pEarlyGate100 via the Gateway LR reaction [27]. The resulting plasmid 35S::*AtPFA-DSP4* was introduced into *Agrobacterium tumefaciens* strain EHA105 by electroporation. Transformation mediated by *A. tumefaciens* was performed as described [28]. The transgenic plants were selected by spraying 1-week-old seedlings with 0.01% Glufosinate (Basta).

### Examination of transgenic plants and knockout mutants

Transgenic rice plants generated with the overexpression and interference recombinant vectors were examined by RT-PCR using two *OsPFA-DSP2* gene-specific primers, 5′-ATGCAGCTGGAGATTTCG-3′ and 5′-TTAACACTGTGAGGCCGTC-3′. The expression level of the *OsPFA-DSP2* gene was assayed by quantitative reverse transcription polymerase chain reaction (qRT-PCR) using the *OsPFA-DSP2* sequence-specific primers, 5′-CCAGTTCGGTATTGACGG-3′ and 5′-TGAGTGCTTCTCGGATTT-3′. The expression level of the rice *β-actin* gene was assayed with actin-specific primers and used to standardize the RNA sample for each qRT-PCR. *β-actin* primers were 5′-GGTATTGTTAGCAACTGGGATG-3′ and 5′-GATGAAAGAGGGCTGGAAGA-3′, respectively. The *OsPFA-DSP2* mutant was identified from a rice mutant collection (International Rice Functional Genomic Consontium; http://irfg.irri.org) harboring a *Tos17* insertion into the 2nd intron of Os02g53160 (seed stock number NG8341) by PCR with the gene-specific primers, 5′-ATTGTTAGGTTGCAAGTTAGTTAAGA-3′ and 5′-GCATTTTGCTCAAACAGGGT-3′, and the *Tos17*-specific primer TAIL13, 5′-GAGAGCATCATCGGTTACATCTTCTC-3′. The *atpfa-dsp4* mutant which obtained from the Arabidopsis Biological Resource Center(ABRC)at Ohio State University (stock name: salk_016876, named as *atpfa-dsp4* mutant) was identified by PCR using the primers: 5′-ATTCCCCAAAACTTCTGA-3′ and 5′-TCTACAACCATCCGATCC-3′. The overexpression in transgenic Arabidopsis was quantified using RT-PCR with the primer pair 5′-AGTCGACATGACGTTAGAGAGTTAC-3′ and 5′-CGGATATCTCAGTAATCAATAGTATT-3′. The expression profile of AtPFA-DSP4 was detected by qRT-PCR using two sequence-specific primers, 5′-TGAGGCATATCCAGAGGT-3′ and 5′-CTACAAGACATCCCGTCC-3′. The expression of the arabidopsis ubiquitin 4 (UBQ4) gene was detected with UBQ-specific primers (5′- GCTTGGAGTCCTGCTTGGACG -3′ and 5′- CGCAGTTAAGAGGACTGTCCGGC -3′) and was used to standardize the sample for each qRT-PCR reaction.

### Pathogen culture and plant infection

*Magnaporthe oryzae* strain Z1 was cultured on PDA medium for 5 days, then transferred to spore production medium and cultured for 24 h at 24°C with 16 h light/8 h dark. Conidia were suspended in sterilized distilled-water at a concentration of ∼3–5×10^5^ conidial/mL. Seedlings at the three-to five-leaf stage were infected with *M. grisea* by the spraying and immersed infection method [17], [29]. Transgenic rice plants after inoculation were initially cultured for 24 h in the dark at 25°C with humidity >95%, followed by the same conditions for 6 days with 16 h light/8 h dark, after which they were observed for disease reaction phenotype. Disease was scored by measuring the lesion area at 7 days after infection. For all the disease evaluations, the mock-inoculated control was treated under identical conditions.

*Arabidopsis* plants were grown in a chamber with a 12 h light/12 h dark photoperiod at 22°C for 24 days before bacterial inoculation. Bacterial infections and growth assays with *Pst* DC3000 were described previously [30]. *Pst* DC3000 was cultured overnight at 28°C in King's B medium supplemented with 50 µg rifampicin/mL. The cells were pelleted, washed, resuspended, and diluted in H_2_O to a concentration of 10^5^ cfu/mL. Arabidopsis leaves were syringe-inoculated. Plant leaves were harvested at the indicated time for ROS detection, F_v_/F_m_ measure and bacterial counting. Bacterial growth was assessed by plating a dilution series of leaf discs ground in H_2_O on King's B plates containing 50 µg rifampicin/mL. The colony-forming units (cfu) were counted after incubation at 28°C for 2 days. Each data point is shown as triplicates.

### Gene expression analysis

To examined the influence of rice blast infection on resistance gene (*OsPR1a* and *OsPR5*) expression, RNA was extracted from leaves with TRIzol reagent (Invitrogen, USA), and cDNA was synthesized by reverse transcription using Prime Script RTase (Takara, Japan). For qRT-PCR assays, SYBR Green I (Takara, Japan) was added to the reaction system and run on a LC480 real-time PCR detection system according the manufacturer's instruction (Roche, Switzerland). Data were analyzed using optical monitor software (Roche). The expression level of the rice *β-actin* gene was used to standardize the RNA (20 ng) sample for each qRT-PCR. The assays were repeated at least three times, with each repetion having three replications; similar results were obtained in repeated experiments. The SD was calculated for each data point. The sequence-specific primers were as follows: *OsPR1a*, 5′-CGTCTTCATCACCTGCAACTACTC-3′ and 5′-CATGCATAAACACGTAGCATAGCA-3′; *OsPR5*, 5′-CGCTGCCCCGACGCTTAC-3′ and 5′-ACGACTTGGTAGTTCTGTTGC-3′; *β-Actin*, 5′-GGTATTGTTAGCAACTGGGATG-3′ and 5′-GATGAAAGAGGGCTGGAAGA-3′.

### Expression pattern analysis

Total RNA was extracted from different tissues of wild-type rice using TRIzol reagent (Invitrogen, USA). The cDNA was synthesized using Prime Script RTase (Takara, Japan) according to the manufacturer's protocol, and the expression of *OsPFA-DSP2* was detected using SYBR Premix Ex Taq (Takara, Japan) with LC480 (Roche, Switzerland). The transcript levels of *OsPFA-DSP2* were normalized to the transcript levels of β-actin. Each data point had three replicates, and the experiments were repeated twice. The results from the two experiments were consistent, and those from one set of experiments are shown.

The promoter of the *OsPFA-DSP2* gene (1,750 bp DNA fragment upstream of the translation start site) was predicted by Osiris (http://www.bioinformatics2.wsu.edu/cgi-bin/Osiris/cgi/home.pl) and amplified with primers 5′-GGGTCTGCTGCACTATACTGG-3′ and 5′-AAAATCCTCCTCTTGGGCG-3′ from genomic DNA of wild type rice, and cloned into the vector pCXGUS-P, which carried the reporter gene GUS (*β*-glucuronidase) [40]. Rice tissues at different growth stages were stained and observed using a light microscope (Leica, Germany); histochemical staining was performed according to a published method [31].

### Subcellular localization of OsPFA-DSP2

The full-length cDNA sequence of *OsPFA-DSP2* was amplified from wild type rice with the primers 5′-GGGTCTGCTGCACTATACTGG-3′ and 5′-AAAATCCTCCTCTTGGGCG-3′, and inserted into pUC-GFP vector. The recombination vector was transiently expressed in rice protoplasts isolated from rice 9-day-old seedlings as described [32]. Cell nuclei were stained with 100 µg/mL 4′,6-diamidino-2-phenylindole (DAPI) for 15–30 minutes. Fluorescence was observed using an Olympus fluorescent microscope and visualized with Olympus DP2-BSW software.

### Detection of H_2_0_2_ by DAB staining

Rice tissues from seedlings with 3–5 leaves (tillering stage) after inoculation were collected at 2 hpi, and then immersed in DAB solution (1 mg/mL) at pH 3.8 for three hours as described [33] and transferred to the light for 8 h. Arabidopsis tissues were treated in a similar fashion, except the vacuum treatment was reduced to 1.5 h, and then transferred to light for 5 h. Plant tissues was then cleared by boiling for 10 minutes in absolute ethanol, and then photographed with a Canon camera.

## Results

### Expression pattern and subcellular localization of OsPFA-DSP2

**Figure 1:**
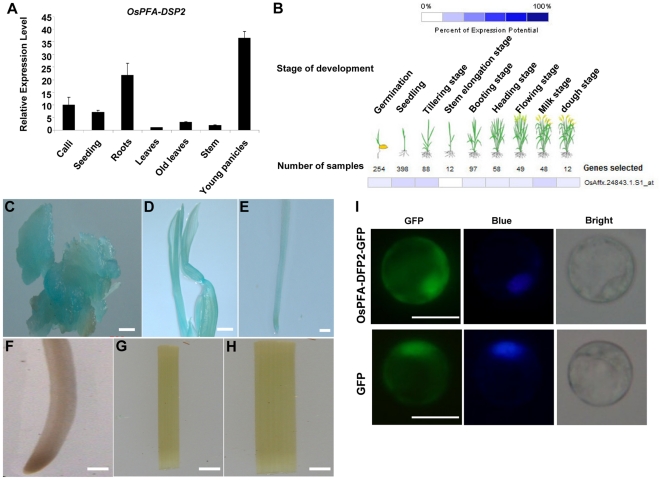
Expression pattern and subcellular localization. (A) Expression profile of *OsPFA-DSP2* in different tissues of wild type rice using qRT-PCR. (B) Expression of *OsPFA-DSP2* at different developmental periods using GENEVESTIGATOR V3. (C–H) Histochemical localization of *GUS* expression in different tissues of transgenic rice carrying the *OsPFA-DSP2* promoter fused to GUS; (C) callus, (D) seedling (five day), (E) young root (five day), (F) old root (flowering stage), (G) stem, and (H) old leaf (flowering stage). (I) Subcellular localization of OsPFA-DSP2 in rice protoplasts by transient transformation, the fluorescence of GFP and DAPI are indicated by green and blue, respectively. Bars = 10 µm.

OsPFA-DSP2 is a 205 amino acid protein that has been annotated as a putative dual-specificity phosphatase (DSPs) belonging to the PFA-DSP subfamily; it contains four conserved motifs characteristic of members of the PFA-DSP subfamily [23]. To determine the expression pattern of *OsPFA-DSP2*, qRT-PCR and GENEVESTIGATOR analysis were carried out, and transgenic plants expressing the GUS gene under control of the OsPFA-DAP2 promoter were generated. Expression of *OsPFA-DSP2* was highest in young panicles, followed by roots, calli, old leaves, stem and leaves (10-days-old) as determined by qRT-PCR (Figure 1A). The probe Os.Affx24843.1.S1_at from *OsPFA-DSP2* was used to analyze an *O. sativa* microarray database (OS_51K: Rice Genome 51K array) in GENEVESTIGATOR V3 [34]. The results of this analysis indicated taht *OsPFA-DSP2* was mainly expressed in seedlings, at the tillering stage and milk stage, and was similar to results of the qRT-PCR assay (Figure 1B).

In order to further demonstrate the expression pattern of *OsPFA-DSP2*, the activitiy of the *Escherichia coli β*-glucuronidase (GUS) gene under control of the *OsPFA-DSP2* promoter region (1750 bp) was examined in transgenic rice plants. A total of 15 independent transgenic lines were analyzed, and all exhibited the same pattern of GUS staining. GUS activity was strongest in calli, young roots and seedling, with very little expression detected in old roots, stems and old leaves (Figure 1C–H). Based on these results, we concluded that *OsPFA-DSP2* is mainly expressed in young tissues.

To investigate the subcellular localization of the OsPFA-DSP2 protein in rice protoplasts, we created constructs for expression of *OsPFA-DSP2* fused to the green fluorescence protein (GFP) under control of the 35S-CaMV promoter. The fluorescence emission of GFP was monitored in rice protoplasts using an Olympus fluorescence microscope. Nuclei were counter-stained with DAPI dye. The GFP fluorescence was distributed in the cytoplasm and nucleus (Figure 1I). OsPFA-DSP2-GFP fusion protein fluorescence was mainly localized to the cytoplasm and nucleus, which could be seen by blue DAPI staining. This result is in agreement with several previous studies that showed protein dual-specificity phosphatases such as AtIBR5, AtMKP2, AtPP2C5, OsIBR5 and OsPFA-DSP1, also localized to the cytoplasm and nucleus when analyzed as protein fusions with fluorescent protein tags [18], [23], [35], [36], [37]. These data indicate that OsPFA-DSP2 may target to both the cytoplasm and nucleus.

### OsPFA-DSP2 is involved in the host response to *M. grisea* infection

**Figure 2:**
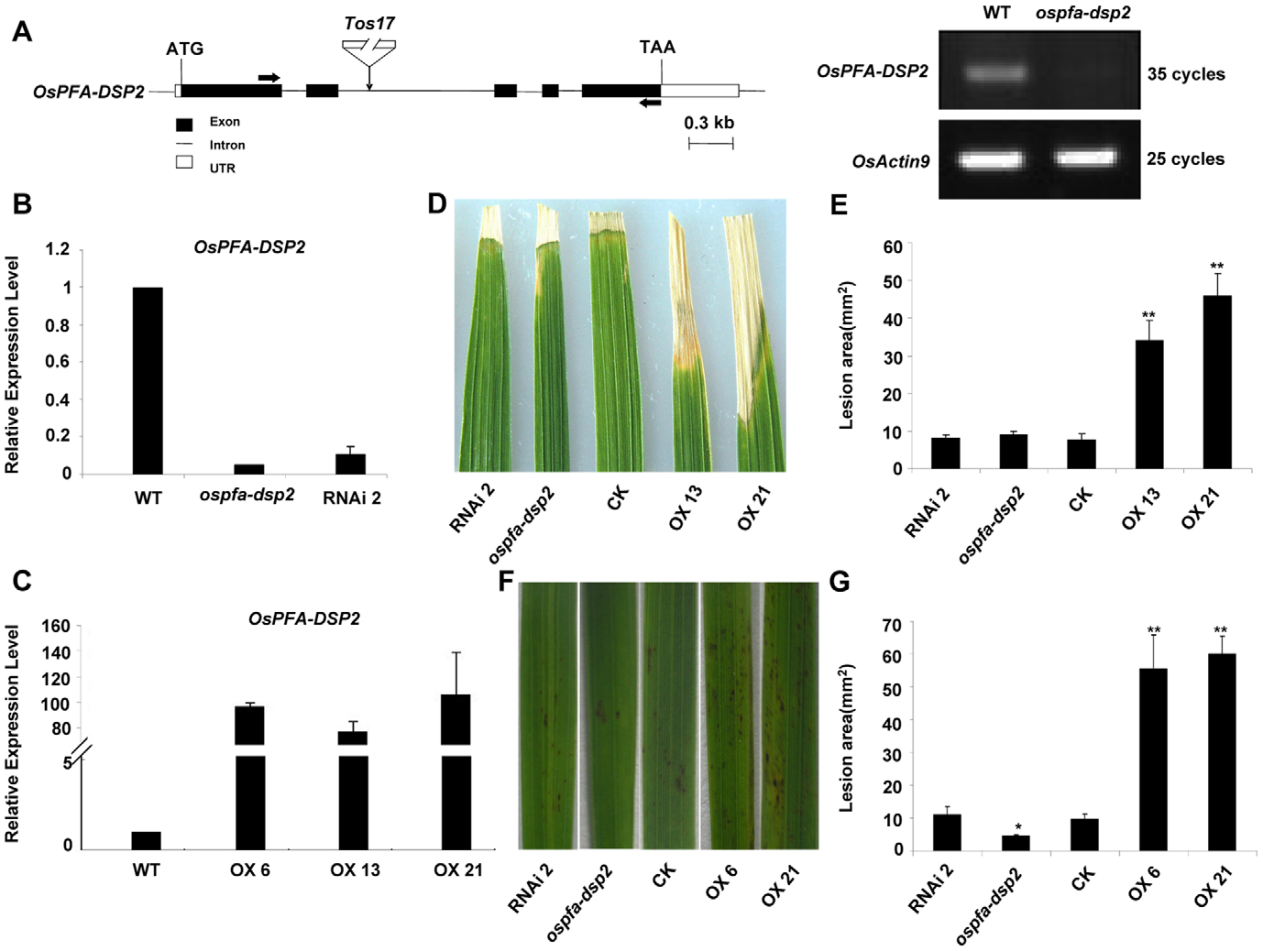
Phenotypes of the *ospfa-dsp2* mutant and *OsPFA-DSP2* transgenic rice plants following *M. grisea* infection. (A) Genomic structure of *OsPFA-DSP2* and transcription level. The retrotransposon *Tos17* was inserted into the second intron; expression of *OsPFA-DSP2* was detected using RT-PCR. (B) and (C) Expression of *OsPFA-DSP2* in wild type rice, mutant, *OsPFA-DSP2*-RNAi and *OsPFA-DSP2*-overexpression plants was detected using qRT-PCR. (D) and (E) Phenotype and lesion area of CK, *ospfa-dsp2* mutant, *OsPFA-DSP2*-overexpression and *OsPFA-DSP2*-RNAi plants at 7 days after inoculation with *M. grisea* conidial suspension. The experiment was repeated with three biological replicates, *P<0.05, **P<0.01.

To demonstrate the function of the *OsPFA-DSP2* gene in rice, the *OsPFA-DSP2* deficiency mutant (seed stock number: NG8142, named as *ospfa-dsp2*) was chosen from the *Tos17* rice database [38]. In this mutant, a retrotransposon (*Tos17*) has inserted into the second intron. Transcription of *OsPFA-DSP2* in the mutant was not detected by semi-reverse transcription polymerase chain reaction (RT-PCR) (Figure 2A). We also generated RNAi transgenic plants to specificly silence *OsPFA-DSP2*; expression of *OsPFA-DSP2* in the mutant and silenced plant (named RNAi2) was suppressed approximately 95% and 89% compared with that in wild type (WT) rice as detected by qRT-PCR (Figure 2B). The expression level of *OsPFA-DSP2* in overexpression lines (named OX6, OX13 and OX21) was increased about 102.5-fold, 83-fold and 112.7-fold, respectively, as compared to WT (Figure 2C). To observe the phenotypes, the F_2_ generation of transgenic rice plants was treated with an *M. grisea* conidial suspension, containing 0.01% Tween 20, by two different methods. Compared with CK, which was transformed with the empty vector (pCXUN-flag vector), the overexpression lines OX13 and OX21 were sensitive to *M. grisea* at 7 days post inoculation (dpi); the lesion area was approximately 4.5-fold and 6-fold larger than that observed on CK (*p*<0.01) (Figure 2D and E). However, the symptoms of the *ospfa-dsp2* mutant and RNAi2 plant did not differ appreciably from those observed on CK at 7 dpi, with the lesion area being similar (Figure 2D and E). Many more hypersensitive response (HR) spots were observed on the surface of OX6 and OX21 leaves than on CK, *ospfa-dsp2* mutant and RNAi2 rice leaves at 7 days after spraying with the *M. grisea* conidial suspension; the lesion area of OX6 and OX21 leaves were ∼5.7-fold and 6.2-fold (*p*<0.01) larger than the area on CK (Figure 2F and G). The number of HR spots on the *ospfa-dsp2* mutant was less than that of CK, and the area of the lesions was reduced by about half (*p*<0.05) (Figure 2F and G). However, the HR spots and lesion area observed on the RNAi2 line was not obviously different from CK (Figure 2F and G). These results indicated that OsPFA-DSP2 could be considered as a negative regulator involved in the plant response to *M. grisea*.

### OsPFA-DSP2 inhibits accumulation of H_2_O_2_ and expression of *PR* genes in response to *M. grisea* infection

**Figure 3:**
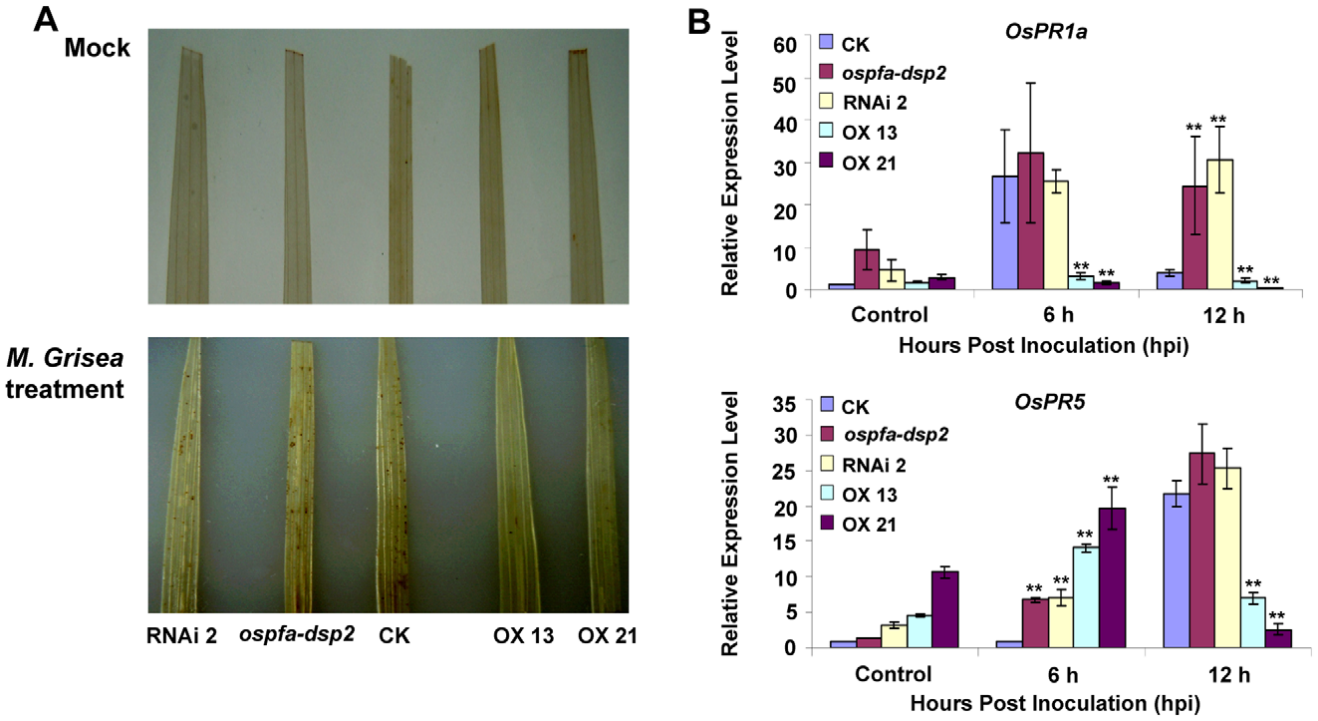
Histochemical detection of H_2_O_2_ by 3^,^3-diaminobenzidine (DAB at 1 mg/mL) staining and detection of defense-related genes using qRT-PCR. (A) Production and accumulation of H_2_O_2_ in leaves at 2 hpi after *M. grisea* infection and mock inoculation (distilled water containing 0.01% Tween 20). (B) Detection of *OsPR1a* and *OsPR5* expression at 6 hpi and 12 hpi following *M. grisea* infection by qRT-PCR. Each experiment consistd of three-independent replicates, given the representative group, **P<0.01.

In order to further study the biological function of phosphatase OsPFA-DSP2, DAB staining was used to detect H_2_O_2_ accumulation in the leaves after infection. Leaves were stained for H_2_O_2_ at 2 hours post inoculation (hpi) with *M. grisea* (3–5×10^5^ conidia/mL containing 0.01% Tween 20) and distilled-water containing 0.01% Tween 20 (control treatment). We observed that the production and accumulation of H_2_O_2_ in rice leaves was not detected by DAB staining in the control, a low level of H_2_O_2_ accumulated in the leaves of CK, the *ospfa-dsp2* mutant and RNAi2 plants at 2 hpi, and H_2_O_2_ accumulation in OX13 and OX21 was minimal (Figure 3A). Also, when compared with CK, the expression level of *OsPR1a* in the OX13 and OX21 plants was reduced by ∼88.2% and 93% at 6 hpi (*p*<0.01), but the expression level of *OsPR1a* in the *ospfa-dsp2* mutant and RNAi2 plant was increased by approximately 1.5-fold at 6 hpi (Figure 3B). The expression profile of *OsPR5* in the *ospfa-dsp2* mutant and RNAi2 plants was also elevated, approximately 1.2-fold compared with CK at 12 hpi, but its expression in OX13 and OX21 was reduced by ∼68% and 88% compared with CK (*p*<0.01) (Figure 3B). These results indicated that OsPFA-DSP2 inhibited the production and accumulation of H_2_O_2_ and suppressed the transcription of *PR* genes, further demonstrating that OsPFA-DSP2 plays a key role in the host response to *M. grisea* infection.

### AtPFA-DSP4 is involved in the host response to *Pst* DC3000 infection

**Figure 4:**
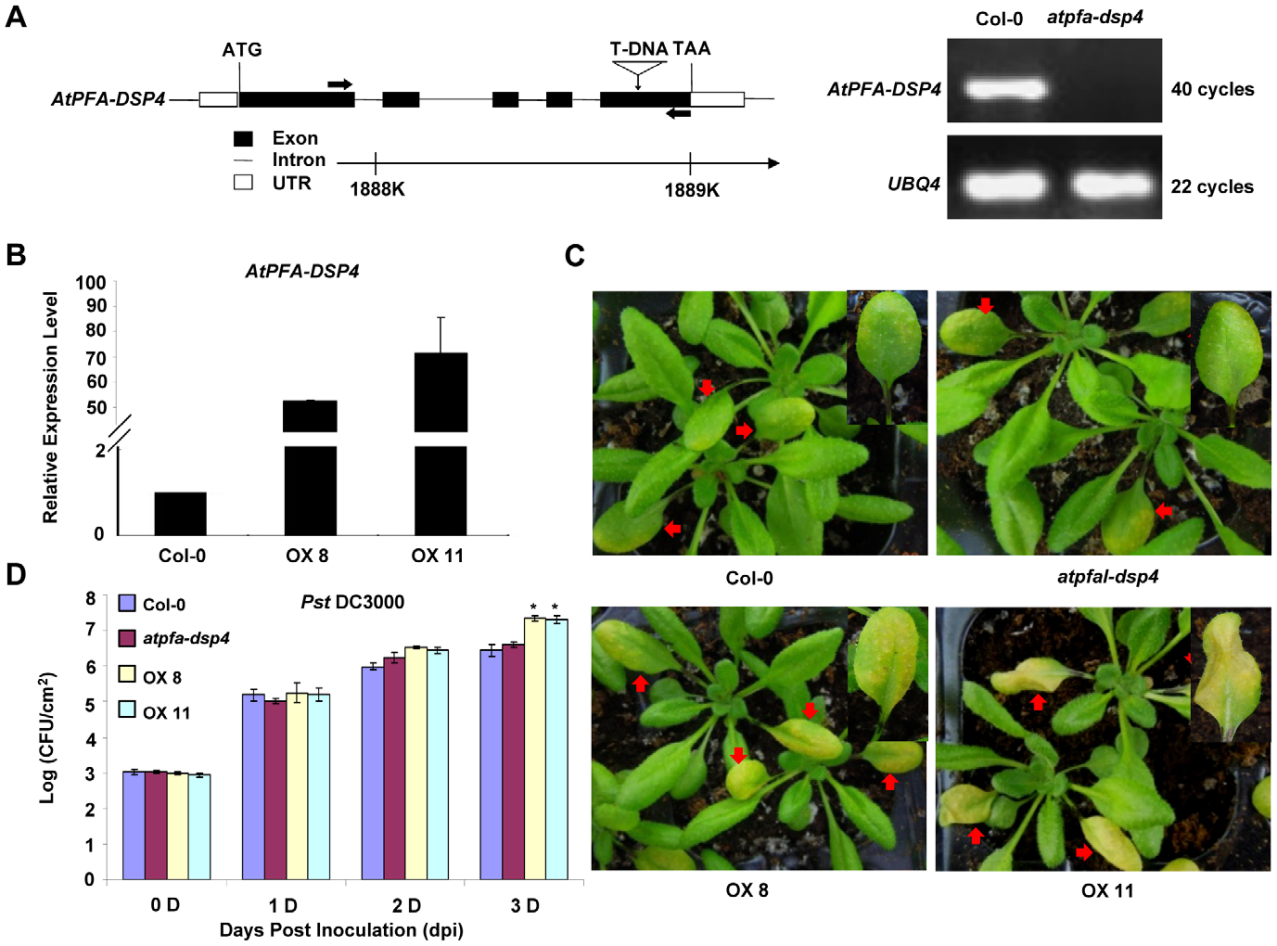
Phenotypes of *atpfa-dsp4* mutant, Col-0 and *AtPFA-DSP4*-overexpression plants following *Pst* DC3000 infection. (A) Genomic structure and transcription of *AtPFA-DSP4* detected using RT-PCR. The T-DNA was inserted into the fifth exon. (B) Expression of *AtPFA-DSP4* in Col-0 and overexpression plants was quantified using qRT-PCR. (C) Phenotype of Col-0, *atpfa-dsp4* mutant, and *AtPFA-DSP4*-overexpression plants at 3 days after *Pst* DC3000 infection. (D) Bacterial counts in leaves of Col-0, *atpfa-dsp4* mutant, and *AtPFA-DSP4*-overexpression plants at different days after inoculation with *Pst* DC3000. The experiment was repeated with three biological replicates *P<0.05.

The homolog of *OsPFA-DSP2* in Arabidopsis is *AtPFA-DSP4*; their protein sequences share a high degree of similarity (identifies = 67%). Because OsPFA-DSP2 is involved in the pathogen response, we were interested to know whether AtPFA-DSP4 has similar function. An AtPFA-DSP4 deficiency mutant (stock name: salk_016876, named as *atpfa-dsp4* mutant) was obtained from the Arabidopsis Biological Resource Center (ABRC) at The Ohio State University. The T-DNA was determined to be inserted in the fifth exon by analyzing the information from database and the gene sequence; transcription of *AtPFA-DSP4* in the mutant was not detected by RT-PCR (Figure 4A). We also generated *AtPFA-DSP4*-overexpressing transgenic plants; two independent overexpression lines, OX8 and OX11, were used in our experiments. The expression levels of *AtPFA-DSP4* in OX8 and OX11 were increased ∼55.5-fold and 74.5-fold compared with Col-0, respectively (Figure 4B). The Arabidopsis plants were treated with a *Pst* DC3000 suspension in distilled water. Compared with Col-0, OX8 and OX11 lines were sensitive to *Pst* DC3000 at 3 dpi; based on color, the lesions on OX8 and OX11 leaves which were injected with the *Pst* DC3000 suspension were more extensive than those observed on Col-0 (Figure 4C). However, the lesion area on the *atfpa-dsp4* mutant was not appreciably different than Col-0 (Figure 4C). We also calculated the amount of *Pst* DC3000 per unit area in leaves infected with *Pst* DC3000. The bacterial cell counts in OX8 and OX11 plants were 1.14-fold and 1.13-fold higher, respectively, than in Col-0 at 3 dpi, but the bacterial cell count in the mutant was not distinct from Col-0 at 3 dpi (Figure 4D). These results indicated that AtPFA-DSP4 is involved in *Pst* DC3000 response and negatively regulated the process.

### AtPFA-DSP4 inhibited accumulation of H_2_O_2_ and photosynthesis in the *Pst* DC3000 response

**Figure 5:**
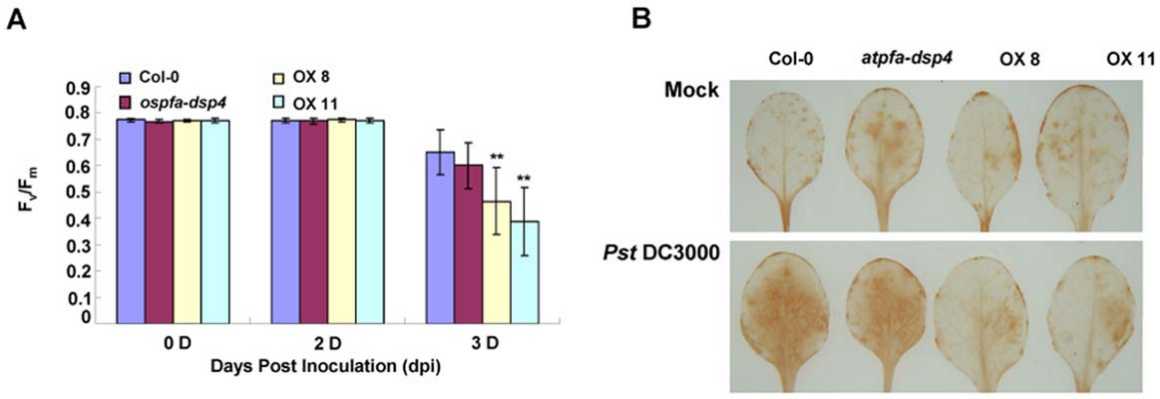
Photosynthesic inhibition and accumulation of H_2_O_2_. (A) The effects of *AtPFA-DSP4* on inhibition of photosynthesis. The photosynthesic capacity of Col-0, *atpfa-dsp4* mutant and *AtPFA-DSP4*-overexpression plants was detected using the IMAGE-PAM system at days zero, two and three following *Pst* DC3000 inoculation. The experiment was repeated with three replicates (plants: n = 10), and averaged, **P<0.01, compared with Col-0 under same treatment condition. (B) Detection of H_2_O_2_ in leaves at 2 hpi after mock (distilled water) treatment and *Pst* DC3000 inoculation. The experiment was repeated with three independent replicates (leaves: n = 4).

We examined the effects of AtPFA-DSP4 on the photosynthesic capability of Arabidopsis after *Pst* DC3000 infection. The results of this study showed that AtPFA-DSP4 inhibited photosynthesis in all plants at 3 dpi, but the inhibition observed in OX8 and OX11 plants was reduced by approximately 29% and 41% compared with Col-0 (Figure 5A). Inhibition of photosynthesis in the *atfpa-dsp4* mutant was not obviously different from Col-0 (Figure 5A). We also detected the generation and accumulation of H_2_O_2_ in the leaves at 2 hpi using DAB staining. The mock treatment (distilled water) did not have an obvious affect on the production and accumulation of H_2_O_2_ in leaves at 2 hpi. However, infection with *Pst* DC3000 resulted in a marked increase in the accumulation of H_2_O_2_ in leaves of Col-0 and the *atpfa-dsp4* mutant, but not in leaves of the OX8 and OX11 plants (Figure 5B). Based on these results, we concluded that AtPFA-DSP4 inhibited photosynthesis and suppressed production and accumulation of H_2_O_2_ during the response to bacterial infection.

## Discussion

Protein phosphatases dephosphorylate activated protein kinases, and play important roles in plant processes such as growth and the reponse to abiotic and biotic stresses [12], [39], [40], [41]. The results presented here show that OsPFA-DSP2 negatively regulates the pathogen response in rice. In the *ospfa-dsp2* mutant and RNAi2 plants, the expression profiles of *OsPR1a* and *OsPR5* were enhanced in the early stages of *M. grisea* infection compared with CK, and the lesion area was similar to that of CK at 7 dpi (Figure 2D–G and 3B). The phenotype of the *ospfa-dsp2* mutant and RNAi2 plants was not obviously different from the CK at 7 dpi, leading us to suggest that the homologous genes exist in rice. The expression of *OsPR1a* and *OsPR5* in OX13 and OX21 plants was decreased compared with CK, and the lesion area was more extensive than in CK, because of overexpression of *OsPFA-DSP2* in rice (Figure 2D–G and 3B). A previous study showed that a mutant deficient in *AtMKP1* expression suppressed the proliferation of *Pst* DC3000 and elevated the resistance to *Pst* DC3000 through the SA-mediated signalling pathway [20]. The function of OsPFA-DSP2, which negatively regulates the response to *M. grisea* infection, was consistent with the function of AtMKP1 [20]. However, rice plants lacking the phosphatase XB15 (a member of the PP2C subfamily) were sensitive to *M. grisea*, and its function was contrary to that of phosphatase OsPFA-DSP2 [17].

We also demonstrated that AtPFA-DSP4 acts as a negative regulator in the host response to *Pst* DC3000 infection. The *AtPFA-DSP4* mutant showed reduced bacterial proliferation and enhanced resistance to *Pst* DC3000, but OX8 and OX11 plants were susceptible to *Pst* DC3000 at 3 dpi (Figure 4C and D). Several studies have shown that AtMKP1 and AtPTP1 negatively regulate the AtMPK3/6 signaling pathway, and act to inhibit biosynthesis of salicylic acid (SA). However, the *AtMKP1* mutant displayed growth defects and enhanced resistance to pathogen *Pst* DC3000 [20], [42]. The function of AtPFA-DSP4 was found to be similar to that of *AtMKP1*, and was contrary to the function of *AtMKP2* [18], [20].

Pathogen infection leads to the generation and accumulation of ROS in plants; H_2_O_2_ is an important component of ROS, and plays key roles in plants, such as regulating growth and development, signal transduction and oxidative damage [4], [43], [44], [45]. The results of our study showed that OsPFA-DSP2 and AtPFA-DSP4 inhibited the production and accumulation of H_2_O_2_ in leaves of overexpression lines in the early stages of infection. Recent studies have reported that endogenous and exogenous H_2_O_2_ inhibited pathogen cell growth, penetration and proliferation at the infection site by inducing PCD [3], [8], [9]. H_2_O_2_ levels in transgenic plants overexpressing OsPFA-DSP2 or AtPFA-DSP4 did not accumulate to control levels, which lead to inactivation of downstream signaling molecules and reduced expression of defense-related genes, such as *PR* genes. *Pst* DC3000 grew and proliferated rapidly in the overexpression lines, which were more susceptible to pathogens. However, because the appearance of black spots on the leaf surface of OX13 and OX21 plants was observed at 7 dpi, we suggest that H_2_O_2_ in the overexpression lines is produced during the later stages of fungal infection, leading to the appearance of HR symptoms (Figure 2F).

The results presented here show that AtPFA-DSP4 and OsPFA-DSP2 (data not shown) inhibited the photosynthesic capacity during the later stages of infection. Pathogen inoculation may result in the production and accumulation of H_2_O_2_, but a certain percentage of H_2_O_2_ is derived from the chloroplast [4], [5], [7], [45]. H_2_O_2_ as a signaling molecule can negatively regulate photosynthesic capacity [46]. Therefore, we presumed that the integrity of chloroplasts was impaired by intense H_2_O_2_ production during the later stages of infection, leading to the observed decrease in photosynthesis in the overexpression lines. However, the production and accumulation of H_2_O_2_ in overexpression plants was not synchronous with the inhibition of photosynthesis. On the one hand, as a first defense system, the burst of H_2_O_2_ is rapid; on the other hand, chloroplast act as a semi-independent organelle, have the ability of auto-repair, resulting in delaying the inhibition of photosynthesic capacity. H_2_O_2_ is an upstream signaling molecule, and is involved in regulating the MAPK cascade [20], [42], [47].

In conclusion, we have demonstrated that two novel homologous genes from rice and Arabidopsis, OsPFA-DSP2 and AtPFA-DSP4, function as negative regulators in the pathogen response through the H_2_O_2_-mediated pathway. However, H_2_O_2_ plays critical roles in different signaling cascades, and which H_2_O_2_-mediated pathway is modulated by OsPFA-DSP2 and AtPFA-DSP4 is not clear. To further understand the functions of OsPFA-DSP2 and AtPFA-DSP4 in response to biotic stress, it will be critical to identify the upstream and downstream effectors which function in the OsPFA-DSP2 and AtPFA-DSP4 signaling pathways, and to demonstrate whether *OsPFA-DSP2* and *AtPFA-DSP4* function in biotic response.
